# Positron Emission Tomography As a Tool for Studying Alcohol Abuse

**Published:** 2008

**Authors:** Panayotis K. Thanos, Gene-Jack Wang, Nora D. Volkow

**Keywords:** Alcohol-related research, alcohol and other drug (AOD) effects and consequences, brain, brain function, brain imaging, positron emission tomography (PET), radiotracers, radioisotopes, (^18^F)-fluoro-2-deoxyglucose (FDG), neurotransmitters, human studies, animal studies

Positron emission tomography (PET) is an imaging technology that measures the concentration, distribution, and pharmacokinetics of radiotracers—molecules that are labeled with short-lived positron-emitting variants (i.e., radioisotopes) of chemical elements naturally found in the body. These radioisotopes can be attached to compounds involved in normal brain function and then injected into the blood stream. For example, radioactive carbon-11 (^11^C) and fluorine-18 (^18^F) can be used to label the sugar glucose, which is the brain’s only energy source, and oxygen-15 (^15^O) can be used to label water molecules, which can help measure blood flow in the brain. The signals emitted by these radiotracers then are measured using specific detectors. For example, for brain measurements, detectors arranged in a ring around the subject’s head collect the data, which are then transferred to a computer and converted into a three-dimensional image of the brain. Because these measurements are noninvasive, the technology allows researchers to track biochemical transformations in the living human and animal body. PET is a highly sensitive method; it measures radioisotope concentrations in the nanomolar to picomolar range (10^−9^ to 10^−12^ M) (Schmidt 2002). Therefore, the technique can be used to label compounds that are of pharmacological and physiological relevance. These radiotracers then can be used to probe neurochemical and metabolic processes at the relevant physiological concentrations without perturbing the system that is measured.

To exert their effects on the brain, alcohol and other drugs (AODs) act on signaling molecules (i.e., neurotransmitters) in the brain as well as on the molecules on the surface of neurons (i.e., receptors) with which the neurotransmitters interact. (For more information on nerve signal transmission, neurotransmitters, and their receptors, see the article by Lovinger, pp. 196–214.) Specific compounds that selectively bind to such receptors, to the molecules that transport neurotransmitters back into cells, and to the enzymes that are involved in the synthesis or metabolism of neurotransmitters can be labeled for use as PET radiotracers. As a result, PET can be used to assess the metabolic and neurochemical actions of AODs and to evaluate the consequences of chronic AOD use (Volkow et al. 1997; Wang et al. 2000; Wong et al. 2003). Since its inception, PET has been used extensively to study the effects of AODs in human and nonhuman primates; however, the recent development of microPET technology has expanded its applications to research in rodents. In addition, increasing numbers of studies are using PET methodology to assess the involvement of genetic variations in individual genes (i.e., polymorphisms) in brain function and neurochemistry. This article specifically summarizes the role of PET as a tool for alcohol neuroscience research. The studies discussed are divided into those that assess the effects of alcohol on brain function (i.e., brain metabolism and cerebral blood flow) and those that assess its effects on neurochemistry.

## PET Analyses of Brain Function

Indicators of brain function, such as cerebral blood flow, glucose utilization, and oxygen consumption, are the most common signals detected in functional brain-imaging techniques. These metabolic signals have been examined in a variety of disorders, primarily through the use of (^18^F)-fluoro-2-deoxyglucose (FDG) as a radiotracer in PET imaging. Thirty-two years after its introduction, FDG still is the most widely used radiopharmaceutical for PET studies. This type of PET imaging allows the noninvasive observation of glucose utilization by different types of brain cells, including neurons and supporting cells known as glial cells (Magistretti and Pellerin 1996). In the brain, the sugar glucose is metabolized to lactate, which is a preferred energy source for neurons. Accordingly, glucose metabolism is a powerful indicator of brain function. FDG–PET imaging has the potential to detect very early brain dysfunction, even before neuropsychological testing yields abnormal results. In addition, the technique can be used to monitor treatment response and the effects of possible therapeutic intervention against the disease.

**Figure 1:**
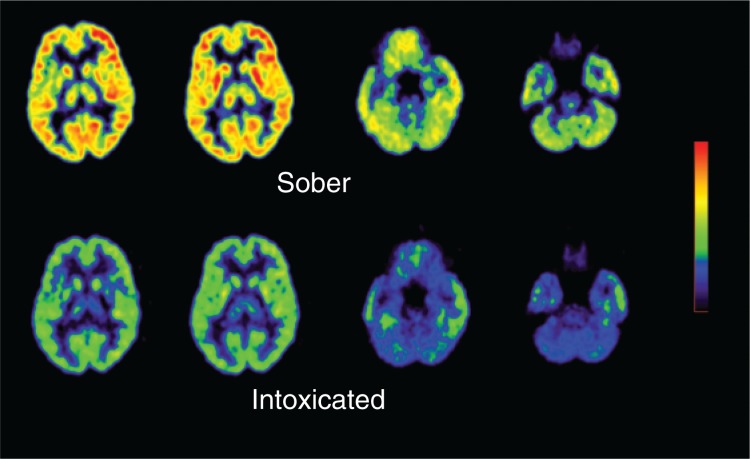
Brain activity during alcohol intoxication. Alcohol drinking markedly reduces brain metabolism.

PET analyses using FDG to measure brain glucose metabolism and radiolabeled water to measure cerebral blood flow have been used to study the acute and chronic effects of alcohol in nonalcoholic control subjects, alcoholics, and people at risk of alcoholism (e.g., children of alcoholics). Other PET studies using FDG have examined alcohol’s toxic effects on neurons (i.e., neurotoxicity) or gender-specific responses to alcohol. The findings include the following:

- Acute alcohol administration markedly reduced brain glucose metabolism throughout the whole brain, including the prefrontal cortex (Volkow et al. 2006) (see figure 1), whereas it increases cerebral blood flow in some brain regions, such as the prefrontal cortex (Volkow et al. 2007). In addition, it was shown that alcoholics displayed both a prefrontal modulation (i.e., reduced brain glucose) in the activity of cells using the neurotransmitter dopamine, combined with a profound decrease in dopamine activity (Volkow et al. 2007). These data suggested that interventions to restore prefrontal regulation and the dopamine deficit could be therapeutically beneficial in alcoholics (Volkow et al. 2007). Moreover, normally, brain metabolism and cerebral blood flow are coupled—that is, areas that show high brain metabolism also exhibit high blood flow and vice versa. Thus, these findings also suggest that alcohol dissociates this metabolic flow coupling.
- A recent FDG–PET study demonstrated abnormally low function of a brain region called the thalamus, which processes and relays information from other brain regions, in alcoholics suffering from acute alcohol-related hallucinations (Soyka et al. 2005).
- Alcoholics and normal subjects respond differently to an acute alcohol challenge, with the alcoholics showing a smaller behavioral response but larger decrease in brain metabolism than normal subjects (Volkow et al. 1993).
- Regional brain metabolic changes in response to treatment with the benzodiazepine medication lorazepam, which, like alcohol, enhances the activity of the neurotransmitter γ-aminobutyric acid (GABA), differed between alcoholic and control subjects. The findings likely indicate altered function of a certain type of GABA receptor (i.e., the GABA–BZ receptor) in alcoholics (Volkow et al. 1995). Indeed, the pattern of regional brain metabolic decrements seen with acute alcohol administration is similar to that observed after acute administration of lorazepam in healthy people, supporting the hypothesis that alcohol and benzodiazepines have a common molecular target for some metabolic effects (Wang et al. 2000).
- Studies measuring brain glucose metabolism or cerebral blood flow documented reduced activity in frontal and parietal cortical regions in alcoholics. This observation is consistent with findings from neuropsychological studies showing that alcoholics have deficits in executive function and attention, which are controlled by these brain areas. Overall, these studies strongly support the concept that alcoholism is associated with damage to the frontal and parietal lobes.
- Several studies have used imaging to probe the recovery of brain function after alcohol withdrawal. These studies found that the alcohol-related decreases in brain glucose metabolism partially recover in abstinent alcoholics, particularly during the first 16 to 30 days after withdrawal (Volkow et al. 1994).

Imaging studies also have addressed the influence of gender on the effects of alcoholism on the brain. It generally is believed that women are more vulnerable to alcohol’s toxic effects than men. However, whereas male alcoholics have consistently shown reductions in brain glucose metabolism relative to control subjects, a PET study using ^18^FDG in 10 recently detoxified female alcoholics reported no differences between alcoholics and control females (Wang et al. 1998). These results do not support the assumption that alcohol has greater toxic effects on the female brain, at least with respect to regional brain glucose metabolism. However, it should be noted that the severity of alcohol use in these female alcoholics was less than that of the male alcoholics previously investigated in PET studies. Therefore, studies in male subjects with moderately severe alcoholism are required to confirm gender differences in sensitivity to alcohol’s effects on brain metabolism.

## PET Analyses of Neurotransmitters and Receptor Binding

PET imaging also has been an effective tool in examining neurotransmitter systems associated with alcohol abuse and alcoholism (for a review of the various neurotransmitter systems affected by alcohol, see Koob 2003; Koob and Le Moal 2008). PET studies have shown that several neurotransmitters appear to mediate alcohol’s reinforcing and addictive effects (Wang et al. 2000). Of these, dopamine is believed to play perhaps the most important role in mediating alcohol’s reinforcing effects by acting on a brain circuit called the mesolimbic dopamine system^1^ (Fowler and Volkow 1998). Researchers have used a plethora of radiolabeled compounds to examine various components of the dopamine system using PET analyses, including the following:

- [^11^C]m-tyrosine, a radiolabeled variant of the amino acid tyrosine, which is the starting material for dopamine synthesis;
- [^18^F]DOPA, a radiolabeled variant of a compound known as 3,4-dihydroxy-l-phenylalanine (l-DOPA), which is an intermediate product in dopamine synthesis;
- A molecule called [^11^C]DTBZ (dihydroytetrabenzine), which helps measure the activity of the vesicular monoamine transporter (VMAT)—a transport protein that helps transport dopamine and other signaling molecules into the vesicles in which they are stored in the signal-emitting (i.e., presynaptic) neuron;
- [^11^C]cocaine, which helps measure the activity of the dopamine transporter (DAT) that shuttles released dopamine back into the presynaptic cells;
- A compound called [^11^C]SCH23390 that helps determine the activity of a certain dopamine receptor, the D_1_ dopamine receptor (D_1_R); and
- A molecule called [^11^C]raclopride, which helps measure the activity of another dopamine receptor, the D_2_ dopamine receptor (D_2_R).

PET imaging studies, as well as postmortem studies of alcoholic subjects, have indicated that D_2_R levels may be involved with alcohol addiction, because the levels of these receptors were reduced in the striatum of the brains of alcoholic subjects (Heinz et al. 2004; Volkow et al. 1996). Additional PET analyses using [^11^C]raclopride demonstrated that higher D_2_R availability in nonalcoholic members of alcoholic families may protect these individuals against alcoholism (Volkow et al. 2006). The data also supported the notion that the low D_2_R levels observed in alcoholics may reflect the effects of chronic alcohol exposures.

Other PET studies have used [^11^C]raclopride to assess changes in dopamine induced by stimulant drugs as a measure of the reactivity of dopamine-releasing cells. This approach is based on the fact that [^11^C]raclopride competes for binding to D_2_ receptors with endogenous dopamine—that is, the more endogenous dopamine is released by the neurons, the less [^11^C]raclopride can bind to the receptor and vice versa. Thus, changes in specific [^11^C]raclopride binding that occur after stimulant administration reflect the relative increases in dopamine induced by the drug. Several studies have revealed a decrease in dopamine release in alcoholic subjects, particularly in the ventral striatum (Martinez et al. 2005; Volkow et al. 2007). In contrast, clinical studies comparing people with a positive family history for alcoholism and people without such a family history did not show differences between the two groups in stimulant-induced dopamine increases in the striatum. These data suggest that the decreased dopamine release in alcoholics is caused by chronic alcohol exposure (Monro et al. 2006). The investigators postulated that the decreased reactivity of the mesolimbic dopamine system in alcoholics could put them at risk of consuming large amounts of alcohol to compensate for deficiencies in this reward pathway.

Investigators also have conducted PET studies using multiple tracers simultaneously to study the relationship between the changes in dopamine activity (as assessed with [^11^C]raclopride) and brain glucose metabolism in the prefrontal cortex (as measured with FDG). These studies demonstrated a negative association between brain glucose metabolism in prefrontal cortical regions (i.e., cingulated gyrus, dorsolateral cortex, and orbitofrontal cortex) and changes in dopamine levels in the striatum (which also contains the nucleus accumbens) of control subjects. Thus, the higher the metabolism in the prefrontal region the lower the changes in dopamine levels. In alcoholic subjects, in contrast, the activity in the prefrontal cortical regions was not correlated with dopamine changes in the striatum (Volkow et al. 2007). These findings suggest that in alcoholics the normal regulation of dopamine cell activity by signals from the prefrontal cortex is disrupted; thus, the decreased dopamine cell activity in alcoholics may represent abnormal prefrontal regulation of the mesolimbic dopamine system.

Another study measured the activity of the vesicular monoamine transporters in alcoholics. This study, which used a radiotracer specific for one type of these transporters (i.e., [^11^C]DTB2), revealed that the levels of this transporter were reduced in the striatum, suggesting that the damaging effects of severe chronic alcoholism on the central nervous system are more extensive than previously considered (Gilman et al. 1998).

PET imaging studies also have been used to examine the role of neurotransmitters known as endogenous opioids in alcohol dependence. Studies using a radiolabeled synthetic opioid pain reliever, [^11^C]carfentanil, showed that the severity of alcohol craving correlated with an increase in a certain type of opiate receptor (i.e., the μ-opiate receptors) in the ventral striatum and, particularly, the nucleus accumbens (Heinz et al. 2005). These findings point to a neuronal correlate of the alcohol craving observed in abstinent alcoholic patients.

Finally, PET analyses have helped examine the neurochemistry underlying the relationship between alcoholism and aggression and, more specifically, whether signal transmission mediated by the neurotransmitter serotonin contributes to this relationship (Brown et al. 2007). The investigators evaluated the density of the serotonin transporter in alcoholic patients who were assessed for aggressive characteristics. The results showed that none of the clinical measures used, including measures of aggression, correlated with serotonin transporter binding in the alcoholic subjects.

## Future Directions

The studies reviewed here reflect the potential of PET as a tool to investigate the alcoholic brain. However, there are additional opportunities for using this technology to investigate the neural underpinnings of alcoholism. For example, PET can be applied to examine the consequences of genetic variations (i.e., polymorphisms), gene modifications, or stem cell procedures on regional brain function in alcoholics.

Other studies have demonstrated the feasibility of using PET to investigate the role of genes in the rodent brain. This development has extended the usefulness of PET in elucidating the role of genes in brain function, aging, and adaptations to environmental and pharmacologic interventions for alcoholism. Technologies to completely disrupt (i.e., “knock out”) or newly introduce (i.e., “knock in”) certain genes in mice have been particularly valuable in elucidating the role of genes and the proteins they encode in normal and pathological behaviors (Avale et al. 2004; Gainetdinov and Caron 2000; Gainetdinov et al. 2001). Other technological advances, such as small-animal PET imaging and microPET technology have rapidly progressed since their introduction (Cherry 1997) and today offer PET images with a resolution of just under 2 mm. Furthermore, the combination of microPET images with images of the same animals obtained using other technologies (e.g., high-field magnetic resonance imaging) has allowed researchers to extend the use of PET imaging studies to rodent models of psychiatric disease (Ding et al. 2004; Rodriguez-Gomez et al. 2007; Thanos et al. 2002, 2008*a*,*b*,*c*,*d*). Thus, microPET has become an effective in vivo imaging tool for noninvasively studying rodent models of alcohol abuse.

## Footnotes

1 This brain circuit primarily involves two brain regions called the ventral tegmental area (VTA) and the nucleus accumbens (NAc). It plays a central role in reward, motivation, and reinforcement. Its activity also is controlled by certain areas of the prefrontal cortex.
